# Nutrition-Related and Self-Rated Health Outcomes Among Lottery-Assigned Residents and Individuals Waitlisted for Subsidized Rental Units in Chinatown, Boston, MA

**DOI:** 10.3390/nu18060878

**Published:** 2026-03-10

**Authors:** Ana Maafs-Rodríguez, Mehreen Ismail, Jennifer Pustz, Laurie Goldman, Peter Levine, Angie Liou, Virginia Chomitz

**Affiliations:** 1 Friedman School of Nutrition Science and Policy, Tufts University, 150 Harrison Avenue, Boston, MA 02111, USA; mehreen.ismail@gmail.com (M.I.); virginia.chomitz@tufts.edu (V.C.); 2 U.S. Census Bureau, 4600 Silver Hill Road, Washington, DC 20233, USA; 3 Department of Public Health & Community Medicine, School of Medicine, Tufts University, 136 Harrison Avenue, Boston, MA 02111, USA; jennifer.pustz@tufts.edu; 4 Office of Student Wellness, School of Medicine, Tufts University, 136 Harrison Avenue, Boston, MA 02111, USA; 5 Graduate School of Arts and Sciences, Urban and Environmental Policy and Planning, Tufts University, 503 Boston Avenue, Medford, MA 02155, USA; laurie.goldman@tufts.edu; 6 Jonathan Tisch College of Civic Life, Tufts University, 163 Packard Avenue, Medford, MA 02155, USA; peter.levine@tufts.edu; 7 Asian Community Development Corporation, 38 Oak Street, Boston, MA 02111, USA; angie.liou@asiancdc.org

**Keywords:** subsidized housing, food insecurity, dietary intake, physical health, mental health

## Abstract

**Background:** Housing is a social determinant of health. In 2015, a lottery assigned low-income families from a waitlist to a new subsidized building (NSB) in Chinatown, Boston, MA. In 2019–2020, we explored associations between housing status (NSB or being on waitlist) and self-rated physical and mental health; household food insecurity (FI); weekly consumption of fruits/vegetables (FV), weekly consumption of soda, and monthly consumption of fast food. **Methods:** Surveys were sent to NSB (*n* = 95) and waitlist (*n* = 2498) households. Logistic and linear regressions explored associations between housing status and outcomes of interest. Models were adjusted for age, sex, Asian background, household size, education, income, employment and distance to the closest food store. **Results:** A total of 138 respondents completed the survey (NSB = 36, waitlist = 102). Groups were demographically similar. In terms of self-reported health, most respondents reported good/better physical health (Waitlist: 62%, NSB: 60%) and good/better mental health (Waitlist: 68%, NSB: 74%). FI was prevalent among both waitlist households (63%) and NSB households (56%). FV intake was similar among NSB households (13.5 times/week) compared to waitlist households (12.8 times/week). The NSB group reported similar soda consumption (1.7 times/week) compared to the waitlist group (2.3 times/week), along with similar fast-food consumption (NSB: 2.7 times/month, Waitlist: 3.7 times/month). We found no statistically significant associations between housing status and outcomes of interest after adjusting for covariates. **Conclusions:** In this small sample, outcomes were not significantly different between groups. Future studies should explore mechanisms through which NSB residence affects nutrition and health, particularly in minority populations.

## 1. Introduction

Housing is a significant social determinant of physical and mental health [1,2,3,4,5,6,7,8,9,10,11,12]. Different frameworks propose theoretical and empirical ways through which housing quality, housing stability, and neighborhood features can impact overall health, mental health, and diet-related outcomes [3,5,6,13]. Overall, these frameworks all acknowledge multiple contextual aspects that directly and indirectly affect health and well-being, such as physical features within buildings, properties, and neighborhoods; financial aspects related to housing cost and stability; and housing experiences such as crowding, perceived safety, comfort, and sense of belonging, among others [1,5,6,13,14,15]. Our understanding of the complex relationship between housing and health is challenged by the lack of consensus on how to define or measure housing-related variables: the often intangible social, psychological, and cultural value that housing provides to individuals and that impacts health outcomes [5,9,13], as well as the difficulty and lack of consensus on the measurement and interpretation of health and nutrition outcomes.

Despite these challenges, a variety of housing interventions are associated with improvements in the health and well-being of individuals. Modification of housing physical features have been effective in reducing and preventing asthma symptoms, respiratory diseases, exposure to prolonged heat waves, and home injuries, among others [9,14]. Additionally, individuals who own a home or have rental vouchers are less likely to suffer from crowding, malnutrition, food insecurity (FI), and housing-related hospitalizations [9,14]. Hence, evidence suggests that policies and interventions that aim to improve housing quality and housing stability could positively impact a variety of health outcomes.

Conversely, while the results were mixed, a review of recent research highlights the negative impact that poor housing quality and housing instability can have on a variety of health outcomes [13]. Housing insecurity, higher housing cost burden, severe rent, high eviction rates, living in public housing, or receiving rental assistance were associated with obesity and diet-related chronic diseases among residents [13]. Similarly, high housing costs, housing instability, poor housing quality, and high foreclosure risk have been associated with a variety of measures, such as less access to primary health care, poor diabetes control, and higher prevalence of hypertension, cardiovascular diseases, infectious diseases, and respiratory illnesses [13,14,16,17,18]. Furthermore, some studies have also linked housing instability and low housing quality with higher distress, anxiety, and depression symptoms, as well as lower self-rated mental health [14,19,20,21]. Unsurprisingly, food security, participation in food assistance programs, and diet quality are also influenced by housing stability. Evidence suggests housing instability is associated with higher levels of household FI and lower diet quality [2,22,23,24,25,26,27].

The evidence about the effect of public housing, subsidized housing, or rental assistance on health and nutrition outcomes is not conclusive, with some differences in outcomes observed among minority populations, particularly when individuals face language and cultural barriers in addition to financial hardships [28,29,30]. Therefore, it is necessary to explore and expand our current understanding of the effects of moving to subsidized housing on residents’ nutrition and health outcomes, particularly among racial minorities.

### Background and Conceptual Framework for the Study

The Asian Community Development Corporation (ACDC) is a non-profit organization located in Boston, MA, which among other services develops affordable housing [31,32]. In 2015, ACDC completed constructions of 95 subsidized rental units in a new building located in Boston’s Chinatown neighborhood and held a housing lottery for these units. Over 4000 households living in the Greater Boston area and beyond applied to the housing lottery. Boston’s Chinatown neighborhood, hereafter referred to as Chinatown, provides a cultural and linguistic hub that addresses many needs of Boston’s Asian communities, particularly the Chinese immigrant community [33]. Organizations and services within Chinatown include but are not limited to the South Cove Community Health Center [34], the largest Asian primary care provider in Massachusetts; the Chinese Progressive Association [35], which works on tenants’ and workers’ rights issues; and the Boston Chinatown Neighborhood Center [36], which offers childcare programs, education initiatives, art programs, and family services. Chinatown also has several authorized locations at which individuals can use or access Supplemental Nutrition Assistance Program (SNAP) and Supplemental Nutrition Program for Women, Infants, and Children (WIC) benefits and services, as well as food pantries, such as the previously mentioned South Cove Community Health Center, South Cove WIC Program main clinic, South End Community Health Center, Tufts Medical Center, and Tzu Chi USA Boston Service Center Food Pantry, among others [34,37,38,39]. Of note, a variety of Asian food markets are also located within Chinatown [40]. Similarly, the neighborhood is located near the Downtown area and has walking-distance access to four subway lines and key bus routes, therefore connecting the neighborhood to other areas around the city [41]. According to the 2020 Census, 47% of the residents in Chinatown are ethnic Chinese, and 67% of the Asian population living there are foreign-born [31].

**Figure 1:**
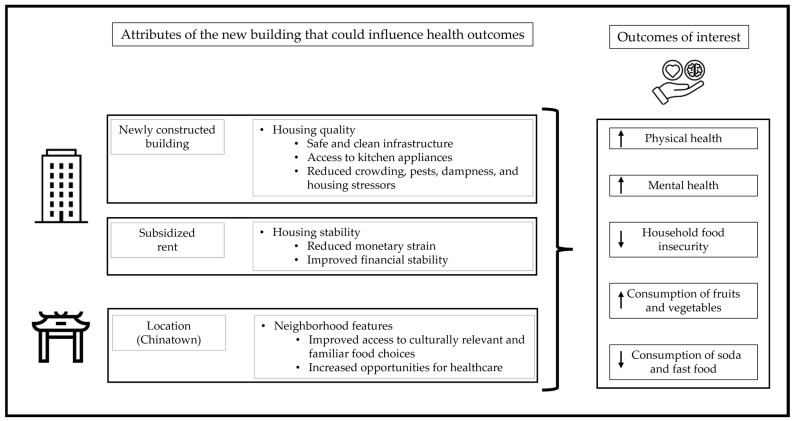
Visualization of attributes of the new subsidized building (NSB). The arrows represent the direction of hypothesized change for each outcome, indicating an overall improvement.

Tufts University researchers from the Health and Housing Study proposed to examine associations between housing conditions and health and well-being after the housing lottery. This situation provided a rare opportunity to explore the association between relocation to a recently built subsidized housing building in an ethnic enclave and residents’ well-being. The present analysis serves as a descriptive pilot study to examine specific health-related outcomes among residents of the new subsidized building (NSB) compared with people who applied to the lottery but remained on the waitlist. Figure 1 shows a visualization of how the combined housing and neighborhood attributes of the NSB (combining physical attributes, financial aspects, and neighborhood characteristics) could influence the health and well-being of NSB residents.

The specific research questions addressed in this descriptive pilot study were: what is the association between housing status (indicated as living in the NSB in Chinatown or being on the waitlist) and (1) self-rated physical health, (2) self-rated mental health, (3) household FI, and (4) weekly consumption of fruits and vegetables, weekly consumption of soda, and monthly consumption of fast food. While our study design was not intended to assess causality and whether or to what extent each of the attributes in Figure 1 relates to our outcomes of interest, we hypothesized that NSB residents would experience better health outcomes compared with individuals on the waitlist due to the collective experience of moving to a new building in Chinatown.

## 2. Materials and Methods

### 2.1. Study Design and Setting

This was a cross-sectional pilot and descriptive study, conducted four years after the housing lottery took place. In 2019–2020 the study team contacted households that had participated in the lottery, including those still on the waitlist and those who had moved to the NSB.

### 2.2. Study Population

An invitation to complete a Qualtrics (Provo, UT, USA) online survey (Supplementary Table S1) with a QR code was sent to the 95 households in the NSB and to 2498 households on the waitlist via mail. Of note, individuals who joined the subsidized housing waitlist were living in different parts of the Greater Boston Area. After the lottery took place, households remained on the waitlist for new housing opportunities to be available.

For this study, respondents were also invited to call study personnel to request a hard copy of the survey or have it administered over the phone. Onsite in-person survey administration was also available to residents in the NSB. The survey was available in English, Chinese, and Spanish and open to only one adult member (≥18 years old) per household. The survey was wide-ranging and assessed respondents’ demographic characteristics, housing and living conditions, neighborhood access and conditions, civic engagement and neighborhood satisfaction, health, diet, and nutrition. For this study, we only focused on survey items related to our variables of interest. All respondents received a gift card as compensation. The survey took approximately 30 min to complete. Both the survey and study protocol were approved by Tufts University Institutional Review Board.

Excluding observations that were duplicates, the initial sample included 136 respondents (100 from the waitlist and 36 from the NSB). Participants who did not answer any of the questions for the dependent or independent variables were excluded from the analysis. The number of missing values in relevant variables ranged from 0 to 9. The final analytical sample included 130 respondents (95 from the waitlist and 35 from the NSB).

### 2.3. Dependent Variables

To fulfill the aims of the present study, the seven dependent variables were assessed as follows:

Self-rated physical and mental health (S-RPH and S-RMH): Two questions on S-RPH and S-RMH were taken from the MOS 36-Item Short-Form Health Survey, developed by Ware and Sherbourne and used in United States (U.S.) nationally representative surveys, including populations of Asian background [42]. Respondents were asked to describe their physical and mental health, separately, by selecting one out of five options: excellent, very good, good, fair, or poor. In agreement with previous literature and considering the study sample size, a binary variable was created, grouping responses as good or better S-RPH or S-RMH, and fair or poor [28]. Of note, these questions were translated literally from English, potentially failing to account for cultural aspects that might influence respondents’ interpretations of terms or the likelihood to report poor-to-fair self-rated health, such as acculturation, gender and age [43,44]. However, U.S. estimates indicate that mental health is an issue of concern for the Asian population, particularly those that have immigrated to the U.S., and self-rated measures have been used in this population [44].

Household food insecurity (FI): Two questions from the U.S. Household Food Security Survey Module were used to determine household FI [45,46,47]. The combination of selected survey questions has been used as an effective and validated screener for household FI, with a sensitivity of 96.4% and specificity of 94.5% [47]. For both questions, respondents were asked to indicate how frequently they had experienced the situation referred to in each question, in relation to food eaten in the household during the previous 12 months (Refer to Supplementary Materials). A binary variable was created to indicate household FI by combining responses to these two questions. A response of ‘often true’ or ‘sometimes true’ to either survey question was considered as affirmative and an indication of FI [45]. Of note, certain terms and concepts used in the validated U.S. Household Food Security Survey Module may be interpreted differently depending on cultural context [48]. Using a literal English-to-Chinese translation, pilot studies have found the prevalence of FI to be more than twice as high among Chinese-speaking respondents compared to respondents from other subpopulations [49].

Weekly consumption of fruits, vegetables, and soda: Questions about usual dietary intake were from the Health Behaviors Section of the California Health Interview Survey (CHIS) [50]. Three questions were asked about the number of times that the respondent had consumed fruits, vegetables, and soda during the past four weeks. Participants could answer either by day, week, or month. We converted all responses to reflect weekly intakes by multiplying reported daily consumption by seven and dividing reported monthly consumption by 4.33 [50]. If respondents entered different amounts for multiple reference periods, all values were converted to weekly intakes and the mean value was obtained. For the purposes of the present analysis, weekly consumption of fruits and vegetables was summed. An implausible value that affected the regression stability was removed from this sub-analysis.

Monthly consumption of fast food: The survey included one question to assess monthly consumption of fast food. No transformation took place, and this variable was continuous.

### 2.4. Independent Variable

The main predictor in all study questions was housing status (NSB versus waitlist).

### 2.5. Covariates

The covariates included age, sex, race, education, household income, employment status, household size, presence of a child younger than 5 years in the household, distance to the nearest food store or market, and report of falls at home in the previous year. The literature identifies these variables as sociodemographic or environmental factors associated with nutrition or health-related outcomes and housing status [2,23,30,51,52]. Considering the small sample size and response distributions, relevant data transformations included treating all covariates as binary variables, except age and household size.

Additional considerations for some covariates included the following:

Race/ethnicity: The survey asked about respondents’ race through five response options (Asian, Native Hawaiian or Pacific Islander, Black or African American, White, or Other), and about Latino/Hispanic ethnicity. The survey did not include a question about Asian background. For the purposes of the analyses, we grouped respondents based on their Asian background, regardless of their Latino/Hispanic ethnicity, categorizing them as having an Asian or Non-Asian background. From this point forward we will use the term Asian to describe this racial background categorization.

Use of food safety net programs: The survey included three questions that asked respondents whether they had received assistance from SNAP, WIC, or a food pantry during the past 12 months. A binary variable was created by combining these three questions and was included as a covariate due to its association with household FI and dietary intake [53].

### 2.6. Statistical Analyses

Descriptive statistics of the sample were assessed by housing status. For continuous variables, we estimated means and assessed differences by housing status using analysis of variance. For categorical variables, we estimated proportions and assessed differences between housing status groups by using chi-square tests.

Multivariable logistic regression models were estimated to determine the odds ratio of reporting good or better S-RPH, good or better S-RMH, and household FI based on housing status. Multivariable linear regression models, with robust standard errors, were estimated to explore the association between housing status and weekly consumption of fruits and vegetables, weekly consumption of soda, and monthly consumption of fast food. All analyses were performed using Stata 15.1 (College Station, TX, USA) [54]. Statistical significance was defined as a *p* value < 0.05.

## 3. Results

The majority of the study sample identified as female (71%) and having Asian background (60%), with an average age of 49 years (Table 1). Across most demographic characteristics, there were no statistically significant differences between housing status groups, except that respondents living in the NSB were more likely to have a child under 5 years in the household (44% versus 22%). Across all respondents, household size averaged about three members, the majority did not have young children in the household (72%), had a household income of less than $40,000 (71%), had a high school diploma or less (55%), and were employed (88%). The majority of respondents across both housing status groups had not fallen at home during the past year (84%). Additionally, the vast majority of respondents reported being within a 20 min walk of the closest food store or market (90%).

There were no statistically significant differences in S-RPH or S-RMH, or in nutrition-related outcomes between housing status groups (Table 2). Most respondents in the overall sample reported good or better S-RPH (61%) and S-RMH (70%). Overall, 61% of all respondents lived in households that experienced FI in the past year. On average, respondents consumed fruits and vegetables 13.0 times per week, soda 2.1 times per week, and fast food 3.4 times per month. Consumption of these foods and beverages was similar between housing status groups.

The overall predictability of the adjusted model with S-RPH as the outcome was statistically significant (Table 3). Holding covariates constant, there was no statistically significant difference in the odds of reporting good or better S-RPH across housing status groups. Older age and experience with a fall at home in the past year were statistically significantly associated with lower odds of reporting good or better S-RPH. Considering mental health (S-RMH) as the outcome, there was no statistically significant difference in the odds of reporting good or better S-RMH across housing status groups. Larger household size was statistically significantly associated with lower odds of reporting good or better S-RMH.

The overall predictability of the adjusted model with household FI as the outcome was statistically significant (Table 3). There was no statistically significant difference in household food insecurity by housing status group, holding covariates constant. Self-reporting as having Asian background was associated with statistically significantly lower odds of household FI.

There were no statistically significant differences in weekly consumption of fruits and vegetables, weekly consumption of soda, or monthly consumption of fast food by housing status group, holding covariates constant (Table 4). Irrespective of housing status group, the only statistically significant predictor in the model for weekly consumption of fruits and vegetables was income, where respondents with household income above $40,000 consumed fewer fruits and vegetables compared to lower-income respondents.

## 4. Discussion

This cross-sectional study explored associations between housing status and health- and nutrition-related outcomes, in individuals who were randomly allocated through a lottery system to live in a NSB compared with individuals still waitlisted for subsidized housing. We hypothesized that living in a newly constructed subsidized apartment building in the vibrant Chinatown neighborhood would be associated with better health and nutrition outcomes through the collective experience of improved housing quality, housing stability, and neighborhood features.

The majority of respondents from both housing groups reported good or better S-RPH (62% from the waitlist, and 60% from the NSB) and S-RMH (68% from the waitlist, and 74% from the NSB). The likelihood of reporting good or better S-RPH and S-RMH was not significantly different between respondents in the NSB or on the waitlist. These results perhaps demonstrate the relatively distal relationship of housing stability, housing quality, and neighborhood characteristics on the more immediate physical and mental health concerns of individuals faced with aging, falls, pests, and the stress of crowded conditions or housing insecurity. Other research suggests that these relationships and housing conditions are very complex and multifaceted, with studies showing mixed results. A study by Gu and Ming conducted in China found that living in high-quality housing was associated with lower self-rated health scores [13]. A study by Ou et al. showed, in contrast, that housing instability was associated with lower self-rated health scores, especially in Latino populations. In Ou et al.’s study, the authors explored in more detail the environmental and social factors that could be driving this association, such as neighborhood characteristics, and cultural stressors (i.e., immigration status), and they also noted the need for more research on this complex issue, particularly for minority groups [28]. Furthermore, questions about S-RPH and S-RMH were translated into Chinese and Spanish, but without taking cultural values into account. Thus, the information gathered may lack the nuance or adaptation necessary to appropriately interpret the results. For example, the translated survey did not account for cultural differences in how populations of Asian background report poor or fair self-rated health or how barriers to healthcare might affect their perception of their own mental and overall health [43,44]. Our results support the collection of qualitative information and a mixed methods approach to help understand the respondents’ responses and the pathways from the NSB residence to residents’ S-RPH and S-RMH.

The adjusted regression model for household FI did not show statistically significant results by housing status. An increased prevalence of household food insecurity among households in unstable housing has been reported previously [16,22,23]. In the present study sample, 63% of respondents from the waitlist reported FI, which is higher than what Kushel et al. found in a sample of *n* = 16,651 from the National Survey of America’s Families (24%) [22], and higher than what a recent study by St-Germain et al. found in low-income renters (28.5%) [23]. Overall, in our analytic model respondents of Asian background were significantly less likely to report being food insecure irrespective of their housing status. The new building in Chinatown provided full kitchens with working appliances and pest-free living conditions that are conducive for storing and preparing food that could be bought more economically in greater quantity and in a less prepared state that is less costly. Not accounting for cultural differences in the conceptualization of hunger and dietary practices, along with other artifacts related to translation, may introduce bias into food security estimates derived from literal translations of the survey items questions used to assess this outcome. Terms and concepts related to health and diet might be interpreted differently depending on cultural factors [48]. Since our survey was translated literally from English into Chinese, this might have introduced bias to our estimates and associations. Our findings require additional analysis and qualitative research to tease out these relationships and interpretation, with careful consideration for cultural adaptation and sensitivity when evaluating these topics.

For diet-related outcomes, fruit and vegetable intake was similar among NSB households (13.5 times/week) compared to waitlist households (12.8 times/week). In addition, the NSB group reported similar consumption of soda (1.7 times/week) compared to the waitlist group (2.3 times/week), and similar monthly consumption of fast food. NSB households reported 2.7 times per month, and waitlist households reported 3.7 times per month. The linear regression models did not show a significant relationship between NSB residence and diet quality components (fruits and vegetables, soda and fast food). Hence, our study did not affirm our hypothesized model of improved nutrition-related outcomes based on NSB status. There are a number of reasons that could account for these findings, mostly related to the complex and intertwined dimensions of housing that holistically affect health, which our study failed to capture or tease out [5,6,9,13]. Based on the literature, we had hypothesized that NSB status would provide building and apartment infrastructure such as new, functional, and well-equipped kitchens, promoting healthful dietary behaviors through opportunities for food storage and cooking. Since high quality foods are often more expensive, subsidized rent could allow households to prioritize healthier eating, and proximity to appropriate, well-priced, and culturally relevant foods could also facilitate shopping patterns conducive to improved dietary patterns. These pathways are supported by the literature [5,11,14,16,22], yet more research with larger sample sizes and longitudinal measurements is needed to further elucidate how housing status directly and indirectly influences these nutrition-related outcomes.

Interestingly, the only statistically significant predictor in our regression model for weekly consumption of fruits and vegetables was income. We found that respondents with household incomes above $40,000 consumed fewer fruits and vegetables compared to lower-income respondents. This result counters existing literature, as higher income households typically consume more fruits and vegetables [27]. While this could be a true difference in our sample, there are a number of factors that might explain this, related to the high number of individuals who indicated that they preferred not to answer the survey question on income, who did not provide an answer, or who might have misreported their income. However, these results warrant future research.

The present study has limitations. The main ones include the small sample size the low survey response rate for both groups, potentially indicating selection bias; missing values in some variables of interest; and limited generalizability to other populations. Of note, the survey for the present study was sent to households in both groups four years after the lottery took place. While moving into a new subsidized housing situation might take some time to influence health and nutrition outcomes, the time lapse between the original lottery and our study might have introduced confounding factors that we failed to capture, including but not limited to job changes, aging, and life trajectory changes. Assumptions for the linear regression models were not fully satisfied, possibly because of the small sample size. Furthermore, the robust standard errors used in the present study reduced power of the regression models, hence some associations might have been underestimated. Since the survey was self-administered, there might have been some bias in participants’ responses. Another important limitation relates to the survey’s questions about race and ethnicity, which failed to disaggregate Asian background into subgroups. Additionally, differences in reference periods across outcomes should be noted. Although all questions were validated, the two survey items through which household FI was assessed inquire about food at home during the past 12 months. Questions about diet quality were more focused on the recent past and asked about regular consumption during the last 4 weeks, at most. It is relevant to mention that all but two of the 35 NSB respondents had been living in the new building for over a full year at the time the survey took place. All survey items were translated without consideration of cultural nuances in how populations of Asian background interpret the terms from those items. While cultural adaptation and validation of culturally relevant surveys was beyond the scope of this paper, this failure might also help explain our null findings.

Our study design did not assess whether or to what extent each of the attributes from Figure 1 was associated with our outcomes of interest. For instance, features of Chinatown may influence our outcomes, such as having easy access to essential healthcare providers, advocacy groups, social services, and specialized food markets. The neighborhood’s central location further enhances this support system by offering residents and visitors convenient, walkable access to diverse nutrition programs and all major city transit lines. Further, receiving a rent subsidy may also explain the outcome, and receiving a cash equivalent to the rent subsidy might even yield the same positive outcomes. Due to the cross-sectional nature of our study, our findings serve as descriptions that can inform future research. While our study did not find statistically significant associations between housing status and health- and nutrition-related outcomes, it serves as a pilot and exploratory study, and as proof of concept for future larger-scale research to explore these relationships. Furthermore, the use of mixed methods that include qualitative approaches could help elucidate the many and complex pathways through which housing and neighborhood shape health behaviors.

## Figures and Tables

**Table 1:** Demographics and descriptive characteristics of respondents by housing status.

	Total	Waitlist (*n* = 95)	New Subsidized Building (*n* = 35)	*p*-Value
Age, years mean (SE)	*n* = 130			
	49 (1.2)	49.8 (1.4)	47 (2.3)	0.33
Household size, members mean (SE)	*n* = 130			
	2.8 (0.1)	2.7 (0.3)	2.9 (0.5)	0.41
Child < 5 years old in the household, *n* (%)	*n* = 99			
Yes	28 (28)	16 (22)	12 (44)	0.03
No	71 (72)	56 (78)	15 (56)	
Sex, *n* (%)	*n* = 130			
Female	92 (71)	65 (68)	27 (77)	0.33
Other	38 (29)	30 (32)	8 (23)	
Household annual income, *n* (%)	*n* = 129			
Less than $40,000	92 (71)	71 (75)	21 (60)	0.10
More than $40,000	22 (17)	12 (13)	10 (29)	
Prefer not to answer	15 (12)	11 (12)	4 (11)	
Highest education level, *n* (%)	*n* = 128			
High school or less	70 (55)	52 (55)	18 (53)	0.81
Higher than high school	58 (45)	42 (45)	16 (47)	
Asian Background, *n* (%)	*n* = 129			
Yes	77 (60)	56 (60)	21 (60)	0.97
No	52 (40)	38 (40)	14 (40)	
Employment, *n* (%)	*n* = 128			
Employed	113 (88)	82 (88)	31 (89)	0.95
Unemployed	15 (12)	11 (12)	4 (11)	
Falls at home in the past year, *n* (%)	*n* = 130			
Yes	21 (16)	17 (18)	4 (11)	0.45
No	109 (84)	78 (82)	31 (89)	
Walking distance to closest food store, *n* (%)	*n* = 129			
<20 min from home	116 (90)	83 (88)	33 (94)	0.32
>20 min from home	13 (10)	11 (12)	2 (6)	

SE = Standard Error.

**Table 2:** Self-rated physical and mental health, and nutrition-related outcomes by housing status.

		Total	Waitlist (*n* = 95)	New Subsidized Building (*n* = 35)	*p*-Value
Self-rated physical health, *n* (%)		*n* = 130			
Fair or Poor Physical Health		50 (39)	36 (38)	14 (40)	0.83
Good or Better Physical Health		80 (61)	59 (62)	21 (60)	
Self-rated mental health, *n* (%)		*n* = 129			
Fair or Poor Mental Health		39 (30)	30 (32)	9 (26)	0.50
Good or Better Mental Health		90 (70)	6 (68)	26 (74)	
Food Insecurity in the past 12 months, *n* (%)		*n* = 129			
Yes		79 (61)	60 (63)	19 (56)	0.45
No		50 (39)	35 (37)	15 (44)	
Regular consumption of food items, times (SE)					
Weekly intake of fruits and vegs	*n* = 128	13.0 (0.8)	12.8 (1.0)	13.5 (1.5)	0.76
Weekly intake of soda	*n* = 121	2.1 (0.4)	2.3 (0.5)	1.7 (0.4)	0.48
Monthly intake of fast food	*n* = 125	3.4 (0.7)	3.7 (1.0)	2.7 (0.7)	0.53

SE = Standard Error.

**Table 3:** Association between household status and self-rated physical health, self-rated mental health, and household food insecurity.

	Self-Rated Physical Health		Self-Rated Mental Health		Household Food Insecurity	
	Adjusted Model * (*n* = 125) *p*-Value 0.0108		Adjusted Model * (*n* = 124) *p*-Value 0.0567		Adjusted Model * (*n* = 123) *p*-Value 0.0285	
Independent Variables	aOR (95% CI)	*p*-Value	aOR (95% CI)	*p*-Value	aOR (95% CI)	*p*-Value
NSB residence	0.84 (0.3, 2.1)	0.71	1.68 (0.6, 4.7)	0.32	0.65 (0.3, 1.6)	0.35
Age	0.94 (0.9, 1.0)	0.00	0.98 (0.9, 1.1)	0.26	1.00 (0.9, 1.0)	0.92
Household size	0.76 (0.6, 1.0)	0.08	0.66 (0.4, 0.9)	0.01	1.16 (0.8, 1.6)	0.36
Female sex	0.61 (0.2, 1.6)	0.32	0.84 (0.3, 2.2)	0.72	1.09 (0.4, 2.9)	0.86
Asian background	2.03 (0.8, 5.4)	0.15	1.41 (0.5, 3.9)	0.49	0.23 (0.1, 0.6)	0.00
Household income		0.50 ^±^		0.19 ^±^		0.27 ^±^
<$40,000	Ref		Ref		Ref	
>$40,000	0.71 (0.2, 2.4)	0.57	0.67 (0.2, 2.3)	0.52	1.73 (0.5, 6.2)	0.40
Prefer not to answer	1.99 (0.4, 8.9)	0.36	6.00 (0.6, 55.3)	0.11	0.43 (0.1, 1.7)	0.23
≤Highschool diploma	0.41 (0.1, 1.2)	0.09	0.62 (0.2, 1.8)	0.38	0.65 (0.2, 1.7)	0.38
Unemployment	1.27 (0.4, 4.4)	0.70	1.21 (0.3, 4.4)	0.77	2.8 (0.7, 12.2)	0.16
Falls at home past year	0.27 (0.1, 0.8)	0.02	0.26 (0.1, 0.8)	0.13	--	--
Store < 20 min away	--	--	--	--	0.86 (0.2, 3.3)	0.82
Use of food safety net	--	--	--	--	0.99 (0.4, 2.4)	0.98

aOR Adjusted odds ratios; CI, Confidence interval: NSB, new subsidized building. * Indicates the model’s predictability to be significant (*p* < 0.005). ^±^ Indicates *p*-value determined by a sum of squares test.

**Table 4:** Association between housing status and weekly consumption of fruits and vegetables, weekly consumption of soda, and monthly consumption of fast food.

	Weekly Consumption of F&V		Weekly Consumption of Soda		Monthly Consumption of Fast Food	
	Adjusted Model ^¥^ (*n* = 122) *p*-Value 0.1187		Adjusted Model (*n* = 116) *p*-Value 0.5072		Adjusted Model (*n* = 119) *p*-Value 0.0934	
Independent Variables	*β* (95% CI)	*p*-Value	*β* (95% CI)	*p*-Value	*β* (95% CI)	*p*-Value
NSB residence	2.03 (−1.5, 5.5)	0.25	−0.39 (−1.9, 1.1)	0.60	−1.19 (−3.9, 1.5)	0.38
Age	0.03 (−0.1, 0.1)	0.60	−0.07 (−0.2, 0.0)	0.09	−0.09 (−0.2, 0.0)	0.12
Household size	−0.13 (−1.1, 0.8)	0.78	−0.02 (−0.6, 0.5)	0.94	−0.12 (−1.2, 0.9)	0.83
Female sex	0.29 (−3.5, 4.0)	0.88	−0.05 (−1.2, 1.1)	0.93	0.08 (−1.5, 1.7)	0.92
Asian background	−1.29 (−5.1, 2.5)	0.49	−2.23 (−4.7, 0.2)	0.07	−2.31 (−6.8, 2.2)	0.31
Household income		0.01 ^±^		0.08 ^±^		0.47 ^±^
Less than $40,000	Ref		Ref		Ref	
More than $40,000	−4.92 (−9.6, −0.9)	0.02	−1.47 (−3.9, 0.9)	0.23	1.62 (−2.5, 5.7)	0.44
Prefer not to answer	1.75 (−4.6, 8.1)	0.58	2.73 (−3.3, 8.7)	0.37	8.95 (−5.7, 23.6)	0.23
≤Highschool diploma	−2.13 (−5.9, 1.7)	0.27	0.64 (−2.3, 3.6)	0.67	2.28 (−4.4, 9.0)	0.50
Unemployment	−0.97 (−7.0, 5.0)	0.75	0.65 (−1.2, 2.5)	0.48	−0.76 (−2.3, 0.8)	0.35
Store <20 min away	−0.34 (−4.3, 3.6)	0.86	1.07 (−1.0, 3.1)	0.31	2.15 (−1.5, 5.8)	0.25
Use of food safety net	−1.43 (−4.5, 1.6)	0.36	−0.73 (−2.9, 1.4)	0.50	1.96 (−0.5, 4.4)	0.11

F&V, fruits and vegetables; CI, Confidence interval; NSB, new subsidized building. ^¥^ Indicates model was run without an influential observation which was removed. ^±^ Indicates *p*-value determined by a sum of squares test.

## Data Availability

The data presented in this study are available on request from the corresponding author due to privacy or ethical restrictions.

## Supplementary Materials

The following supporting information can be downloaded at: https://www.mdpi.com/article/10.3390/nu18060878/s1, Table S1: Survey Questions for Dependent Variables.

## Abbreviations

The following abbreviations are used in this manuscript:

CI	Confidence Interval
FI	Food insecurity
NSB	New subsidized building
OR	Odds ratio
SNAP	Supplemental Nutrition Assistance Program
WIC	Special Supplemental Nutrition Program for Women, Infants and Children

## References

[1] Angel S., Bittschi B. (2019). Housing and Health. Rev. Income Wealth.

[2] Bottino C.J., Fleegler E.W., Cox J.E., Rhodes E.T. (2019). The Relationship Between Housing Instability and Poor Diet Quality Among Urban Families. Acad. Pediatr..

[3] Dweik I., Woodhall-Melnik J. (2022). A systematic review of the relationship between publicly subsidised housing, depression, and anxiety among low-Income households. Int. J. Hous. Policy.

[4] Bhat A.C., Fenelon A., Almeida D.M. (2025). Housing insecurity pathways to physiological and epigenetic manifestations of health among aging adults: A conceptual model. Front. Public Health.

[5] Rolfe S., Garnham L., Godwin J., Anderson I., Seaman P., Donaldson C. (2020). Housing as a social determinant of health and wellbeing: Developing an empirically-informed realist theoretical framework. BMC Public Health.

[6] Swope C.B., Hernández D. (2019). Housing as a determinant of health equity: A conceptual model. Soc. Sci. Med..

[7] Novin N., Jones S.S., Cohn E., Parikh N., Zhang D., Yu P.-J., Coleman K., Leon L.D.O., Chiuzan C. (2025). The health effects of housing instability and its association with congestive heart failure. Am. J. Prev. Cardiol..

[8] Rafla-Yuan E., Handunge V.L., White J.J., Castillo E.G. (2024). Housing, Homelessness, and Mental Health. Psychiatr. Ann..

[9] Koeman J., Mehdipanah R. (2021). Prescribing Housing: A Scoping Review of Health System Efforts to Address Housing as a Social Determinant of Health. Popul. Health Manag..

[10] Sims M., Kershaw K.N., Breathett K., Jackson E.A., Lewis L.M., Mujahid M.S., Suglia S.F. (2020). Importance of Housing and Cardiovascular Health and Well-Being: A Scientific Statement from the American Heart Association. Circ. Cardiovasc. Qual. Outcomes.

[11] Maqbool N., Viveiros J., Ault M. (2015). The Impacts of Affordable Housing on Health: A Research Summary. INSIGHTS from Housing Policy Research.

[12] Holtan M.T., Bowen E., Maisel J., Riva M. (2024). Housing for care, connection, and health equity. Health Place.

[13] Gu K.D., Faulkner K.C., Thorndike A.N. (2023). Housing instability and cardiometabolic health in the United States: A narrative review of the literature. BMC Public Health.

[14] Howden-Chapman P., Bennett J., Edwards R., Jacobs D., Nathan K., Ormandy D. (2023). Review of the Impact of Housing Quality on Inequalities in Health and Well-Being. Annu. Rev. Public Health.

[15] Frieden T.R. (2010). A framework for public health action: The health impact pyramid. Am. J. Public Health.

[16] Martin P., Liaw W., Bazemore A., Jetty A., Petterson S., Kushel M. (2019). Adults with Housing Insecurity Have Worse Access to Primary and Preventive Care. J. Am. Board Fam. Med..

[17] Ahmad K., Erqou S., Shah N., Nazir U., Morrison A., Choudhary G., Wu W.-C. (2020). Association of poor housing conditions with COVID-19 incidence and mortality across US counties. PLoS ONE.

[18] Sims K.D., Willis M.D., Hystad P.W., Batty G.D., Bibbins-Domingo K., Smit E., Odden M.C. (2023). Neighborhood Characteristics and Elevated Blood Pressure in Older Adults. JAMA Netw. Open.

[19] Leventhal T., Brooks-Gunn J. (2003). Moving to Opportunity: An Experimental Study of Neighborhood Effects on Mental Health. Am. J. Public Health.

[20] Pomeroy J.M.L., Johnson E., Weinstein A.A. (2021). Subsidized Housing and Health: An Exploratory Study Examining Resident Perspectives on Community Health and Access to Care. J. Health Care Poor Underserved.

[21] Walker D.O.H., Rabelo V.C., Stewart O.J., Herbert D.N. (2024). Social determinants of mental health: The roles of traumatic events, financial strain, housing instability, food insecurity, and commute time. J. Am. Coll. Health.

[22] Kushel M.B., Gupta R., Gee L., Haas J.S. (2006). Housing instability and food insecurity as barriers to health care among low-income Americans. J. Gen. Intern. Med..

[23] Fafard St-Germain A.A., Tarasuk V. (2020). Homeownership status and risk of food insecurity: Examining the role of housing debt, housing expenditure and housing asset using a cross-sectional population-based survey of Canadian households. Int. J. Equity Health.

[24] Zhang B., Wrenn D.H., Joshi J., Jaenicke E.C. (2022). Housing wealth, food spending, and diet quality: Evidence from panel data. Agric. Resour. Econ. Rev..

[25] Lee C.Y., Zhao X., Reesor-Oyer L., Cepni A.B., Hernandez D.C. (2021). Bidirectional Relationship Between Food Insecurity and Housing Instability. J. Acad. Nutr. Diet..

[26] Chang Y., Chatterjee S. (2022). Housing Instability, Food Insecurity, and Barriers to Healthy Eating. Fam. Consum. Sci. Res. J..

[27] Lee S.H. (2022). Adults Meeting Fruit and Vegetable Intake Recommendations—United States 2019. Morbidity and Mortality Weekly Report.

[28] Ou J.Y., Peters J.L., Levy J.I., Bongiovanni R., Rossini A., Scammell M.K. (2018). Self-rated health and its association with perceived environmental hazards, the social environment, and cultural stressors in an environmental justice population. BMC Public Health.

[29] Sanbonmatsu L., Potter N.A., Adam E., Duncan G.J., Katz L.F., Kessler R.C., Yang F., Kling J.R., Gennetian L.A., McDade T.W. (2012). The Long-Term Effects of Moving to Opportunity on Adult Health and Economic Self-Sufficiency. Cityscape.

[30] Harley A.E., Yang M., Stoddard A.M., Adamkiewicz G., Walker R., Tucker-Seeley R.D., Allen J.D., Sorensen G. (2014). Patterns and predictors of health behaviors among racially/ethnically diverse residents of low-income housing developments. Am. J. Health Promot..

[31] Chan P., Gossage A., Ho G., Huang C., Shen Kuo J., Lee S., Lee T., Liou A., Lowe L., Tsoi T. (2020). 2020 Boston Chinatown Master Plan.

[32] Asian Community Development Corporation (2025). https://asiancdc.org/.

[33] Rubin C.L., Chomitz V.R., Woo C., Li G., Koch-Weser S., Levine P. (2021). Arts, Culture, and Creativity as a Strategy for Countering the Negative Social Impacts of Immigration Stress and Gentrification. Health Promot. Pract..

[34] South Cove Community Health Center (2026). https://scchc.org/.

[35] Chinese Progressive Association (2026). https://cpaboston.org/about/.

[36] BCNC (2026). BCNC Strengthening New Immigrant Families. https://bcnc.net/.

[37] Buddhist Tzu Chi Foundation (2026). Tzu Chi USA Boston Service Center Food Pantry.

[38] Mass.gov (2026). SNAP Outreach Partners. https://www.mass.gov/info-details/snap-outreach-partners#:~:text=Table_title:%20Find%20SNAP%20outreach%20partners%20in%20your,Children%2C%20teens%2C%20adults%2C%20families%2C%20and%20seniors%20%7C.

[39] Mass.gov (2026). WIC Information for Participants Locations. https://www.mass.gov/wic-information-for-participants/locations?icons=All&helper=Boston%2C+MA+02115%2C+USA&lat=42.339904&lng=-71.0898892.

[40] Bokksu Market (2025). Where Can You Find the Best Asian Food Markets in Boston?. https://bokksumarket.com/blogs/magazine/where-can-you-find-the-best-asian-food-markets-in-boston?srsltid=AfmBOooWB1satRITbFg2KRseuCcQl6OW1gvxkFCsZyJqJ-O7CHfAjd9x.

[41] Massachusetts Bay Transportation Authority (2026). Routes. https://www.mbta.com/.

[42] Ware J.E., Sherbourne C.D. (1992). The MOS 36-Item Short-Form Health Survey (SF-36): I. Conceptual Framework and ItemSelection. Med. Care.

[43] Kim M.J., Gorman B.K. (2021). Acculturation and Self-rated Health Among Asian Immigrants: The Role of Gender and Age. Popul. Res. Policy Rev..

[44] Kim S.B., Lee Y.J. (2022). Factors Associated with Mental Health Help-Seeking Among Asian Americans: A Systematic Review. J. Racial Ethn. Health Disparities.

[45] Abbasi N., Ghoochani O.M., Ghanian M., Kitterlin M. (2016). Assessment of Households’ Food Insecurity through use of a USDA Questionnaire. Adv. Plants Agric. Res..

[46] USDA Economic Research Service (2026). Food Security in the U.S.—Survey Tools. https://www.ers.usda.gov/topics/food-nutrition-assistance/food-security-in-the-us/survey-tools#household.

[47] Gundersen C., Engelhard E., Crumbaugh A.S., Seligman H.K. (2017). Brief assessment of food insecurity accurately identifies high-risk US adults. Public Health Nutr..

[48] Arteaga I., Wilde P.E. (2023). Measuring Food Security in the United States for More Than 25 years: History, Methods, Findings, and Opportunities. J. Acad. Nutr. Diet..

[49] Kwan C.M., Napoles A.M., Chou J., Seligman H.K. (2015). Development of a conceptually equivalent Chinese-language translation of the US Household Food Security Survey Module for Chinese immigrants to the USA. Public Health Nutr..

[50] Centre for Health Policy and Research (2026). California Health Interview Survey (CHIS). https://healthpolicy.ucla.edu/our-work/california-health-interview-survey-chis.

[51] Ferguson T.S., Younger-Coleman N.O., Mullings J., Francis D., Greene L.-G., Lyew-Ayee P., Wilks R. (2020). Neighbourhood socioeconomic characteristics and blood pressure among Jamaican youth: A pooled analysis of data from observational studies. PeerJ.

[52] Rummo P.E., Guilkey D.K., Shikany J.M., Reis J.P., Gordon-Larsen P. (2017). How do individual-level sociodemographics and neighbourhood-level characteristics influence residential location behaviour in the context of the food and built environment? Findings from 25 years of follow-up in the CARDIA Study. J. Epidemiol. Community Health.

[53] Bruening M., McClain D., Ma M.M., Reifsnider E. (2017). The Role of SNAP in Home Food Availability and Dietary Intake among WIC Participants Facing Unstable Housing. Public Health Nurs..

[54] StataCorp LLC (2025). Stata Statistical Software: Release 15.

