# Supplementary material for: Nutrition-Related and Self-Rated Health Outcomes Among Lottery-Assigned Residents and Individuals Waitlisted for Subsidized Rental Units in Chinatown, Boston, MA
Source: Nutrients. 2026 Mar 10;18(6):878. doi: 10.3390/nu18060878 (PMC13029076; doi:10.3390/nu18060878)
Supplement: Supplementary file 1 [file nutrients-18-00878-s001.zip › nutrients-4156227-supplementary.pdf]

Supplementary Table S1. Survey Questions for Dependent Variables.

Variable	Survey Questions
Self-rated Physical Health	In general, would you say your physical health is:<br>• Excellent<br>• Very good<br>• Good<br>• Fair<br>• Poor
Self-rated Mental Health	In general, would you say your mental health is:<br>• Excellent<br>• Very good<br>• Good<br>• Fair<br>• Poor
Household Food Insecurity	These next questions are about the food and cost of food eaten in your household in the past 12 months.<br>The food that I bought just didn't last, and I didn't have money to get more.<br>• Often true<br>• Sometimes true<br>• Never true<br><br>I couldn't afford to eat well-balanced nutritious meals at home.<br>• Often true<br>• Sometimes true<br>• Never true
Weekly Consumption of Fruits and Vegetables/Weekly Consumption of soda	Think about the foods you ate or drank in the past 4 weeks, including meals and snacks. How many times did you eat each food per day, week, or month? Choose the time frame that makes the most sense for each category. For example, if you have an apple every day, enter 1 under "Per day."<br>• How many times did you eat fruit? Do not count juices.<br>• How many times did you eat any kind of fried potatoes, including French fries, home fries, or hash browns?<br>• How many times did you eat any other vegetables like green salad, green beans, or potatoes? (Do not include fried potatoes)<br>• How often did you drink regular soda or pop that contains sugar? Do not include diet soda.
Monthly Consumption of Fast Food	In the past 4 weeks, how many times did you eat fast food? Include fast food meals eaten at work, at home, or at fast-food restaurants, carryout or drive-through.
